# Modeling Future Conservation of Hawaiian Honeycreepers by Mosquito Management and Translocation of Disease-Tolerant Amakihi

**DOI:** 10.1371/journal.pone.0049594

**Published:** 2012-11-20

**Authors:** Peter H. F. Hobbelen, Michael D. Samuel, Dennis A. LaPointe, Carter T. Atkinson

**Affiliations:** 1 Department of Forest and Wildlife Ecology, University of Wisconsin, Madison, Wisconsin, United States of America; 2 U.S. Geological Survey, Wisconsin Cooperative Wildlife Research Unit, University of Wisconsin, Madison, Wisconsin, United States of America; 3 U.S. Geological Survey, Pacific Island Ecosystems Research Center, Hawaii National Park, Hawaii, United States of America; New Mexico State University, United States of America

## Abstract

Avian malaria is an important cause of the decline of endemic Hawaiian honeycreepers. Because of the complexity of this disease system we used a computer model of avian malaria in forest birds to evaluate how two proposed conservation strategies: 1) reduction of habitat for mosquito larvae and 2) establishment of a low-elevation, malaria-tolerant honeycreeper (Hawaii Amakihi) to mid-elevation forests would affect native Hawaiian honeycreeper populations. We evaluated these approaches in mid-elevation forests, where malaria transmission is seasonal and control strategies are more likely to work. Our model suggests the potential benefit of larval habitat reduction depends on the level of malaria transmission, abundance of larval cavities, and the ability to substantially reduce these cavities. Permanent reduction in larval habitat of >80% may be needed to control abundance of infectious mosquitoes and benefit bird populations. Establishment of malaria-tolerant Amakihi in mid-elevation forests increases Amakihi abundance, creates a larger disease reservoir, and increases the abundance of infectious mosquitoes which may negatively impact other honeycreepers. For mid-elevation sites where bird populations are severely affected by avian malaria, malaria-tolerant Amakihi had little impact on other honeycreepers. Both management strategies may benefit native Hawaiian honeycreepers, but benefits depend on specific forest characteristics, the amount of reduction in larval habitat that can be achieved, and how malaria transmission is affected by temperature.

## Introduction

More than one-third of all U.S. listed bird species occur in Hawaii, and 71 Hawaiian birds have gone extinct since humans colonized the islands [1]. Introduction of avian malaria (*Plasmodium relictum*) and its vector, the Southern House Mosquito (*Culex quinquefasciatus*), are considered primary contributors to population declines of native Hawaiian forest birds [2]. The endemic Hawaiian honeycreepers (Drepanidinae) are particularly susceptible to avian malaria, have one of the highest rates of extinction in the world [3], and many species survive mainly in higher elevation forests that serve as disease refugia [2].

Temporal and spatial dynamics of malaria in Hawaii are driven by seasonal, annual, and elevational weather patterns [4]–[7]. Due to the steep slopes of many Hawaiian Islands, temperature and rainfall changes quickly, creating altitudinal differences in malaria transmission [2], [8], [9] that are characterized by rapid endemic transmission in warm, low-elevation forests to seasonal epidemics in cooler mid-elevations, and little malaria transmission at high-elevations [7]. In Hawaii, both mosquito dynamics and malaria development respond positively to increased temperatures [4], [5], [10]. As a result, global warming is likely to expand the distribution of malaria into high-elevation forests, decreasing the disease-free refuge for Hawaiian honeycreepers [8], [9] and further reducing avian populations.

To reduce future population impacts and potential species extinctions, conservation strategies that reduce the impact of avian malaria on Hawaiian honeycreepers are essential. However, conservation strategies to preserve future populations of Hawaiian birds can be controversial if focused strictly on habitat improvement [11], are likely costly [1], and have considerable uncertainty related to species impacts. Recently, simulation models have been used to evaluate the potential success of different disease mitigation strategies [12], [13], sometimes with differing conclusions that are indicative of the uncertainty associated with both the ecological system and future predictions. Suggested management actions have included captive propagation and translocation of disease resistant or tolerant native birds [11], [14] or reduction of mosquito densities to reduce disease transmission [15], as well as predator control, habitat conservation and restoration [16]. In this paper we evaluate translocation of native disease tolerant Amakihi and reduction in mosquito populations to increase the abundance of native birds.

Common methods for reducing mosquito abundance include reduction of larval habitat, insecticides that increase larval or adult mortality rates, and the introduction of predators on mosquitoes [16], [17]. At a landscape scale, insecticidal and biocontrol can be difficult to apply, in dense, inaccessible forests and may be unacceptable due to potential negative effects on other native invertebrates. However, on the Island of Hawaii, feral pigs (*Sus scrofa*) feed on native tree ferns (*Cibotium glacum*) creating cavities that fill with rainwater. These cavities are an important and ideal habitat for mosquito larvae, especially in mid-elevation forests [16]. The removal of feral pigs may potentially reduce the impact of avian malaria on endemic Hawaiian honeycreepers. Whether this is a useful management strategy likely depends on the reduction in mosquito habitat and therefore the reduction in pig density that is needed to increase honeycreeper populations.

Although most of the native Hawaiian forest birds are highly susceptible to avian malaria [18], native Hawaii Amakihi (*Hemignathus virens*) found in low-elevation forests may have developed malaria-tolerance compared to conspecific birds at mid- and high- elevations [19], [20] (C.T. Atkinson, unpublished data). Although these birds are relatively sedentary compared to other Hawaiian forest birds, it seems likely low-elevation birds will eventually disperse into mid-elevation forests. In addition, translocation of native malaria-tolerant species to new areas has been suggested as a conservation action to facilitate disease tolerance in the native avian community [11], [15]. Individual birds that recover from avian malaria develop immunity to further infection, but remain infectious to susceptible mosquitoes [6], [21]. These chronically infected birds provide the primary reservoir for malaria transmission [6], [18]. Increased abundance of malaria-tolerant Amakihi could therefore increase transmission to other susceptible honeycreepers by providing a larger disease reservoir for infecting mosquitoes. Therefore, prior to management actions, it is essential to evaluate the potential effect of malaria-tolerant Amakihi on other Hawaiian honeycreepers.

Ecological models can provide an indispensable tool for exploring the consequences of alternative management strategies [22] that would be costly or time consuming to test in practice. In particular, modeling of the avian malaria system can provide a tool to account for the spatial and temporal complexity, evaluate the potential outcome of alternative conservation actions to reduce avian malaria, and predict how climate patterns may affect disease dynamics. Samuel et al. [7] derived and tested a model of avian malaria transmission in a community of native Hawaiian honeycreepers. This model accounts for the spatial and temporal variation in temperature and rainfall along the steep slopes of Hawaii, which are important drivers of the vector and malaria dynamics. We used this model to evaluate the potential impacts on native Hawaiian forest birds of 1) reducing mosquito larval habitat and 2) establishment of malaria-tolerant Amakihi from low- to mid-elevation forests on Hawaii. Because conducting large-scale conservation actions or even field experiments is both costly and impractical, we used this computer simulation model to evaluate the potential success of these conservation strategies on disease transmission and avian populations at specific forest sites on the windward side of Hawaii.

## Materials and Methods

All necessary access and collection permits for this study were obtained from the State of Hawaii, Department of Land and Natural Resources and Hawaii Volcanoes National Park.

### Study sites

**Figure 1:**
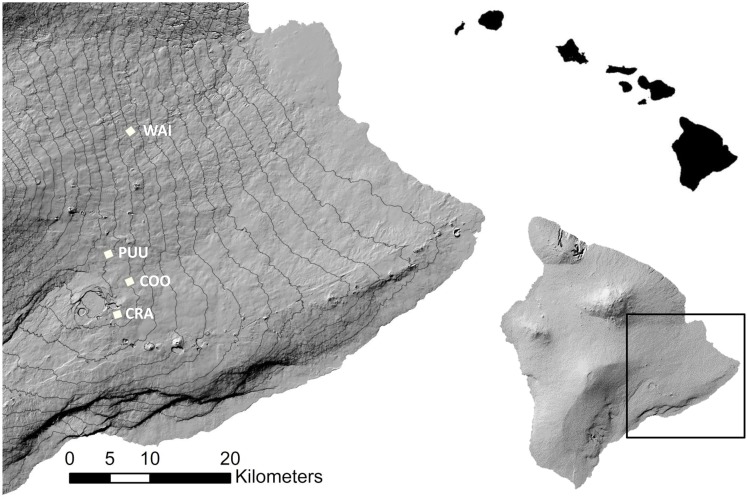
Map of mid-elevation study sites (Crater = CRA, Cooper = COO, Pu'u = PUU, Waiakea = WAI) located on the eastern slope of Mauna Loa and Kilauea Volcanoes on the Island of Hawaii. Elevation contours are 100 meter intervals. See text for additional information on study sites.

We simulated the effect of the larval habitat reduction and establishment of malaria-tolerant Amakihi on avian malaria in honeycreeper populations at four 1-km^2^ study sites with mosquito larval habitat (Table 1) in mid-elevation (885–1247 m) forests in the proximity of Hawaii Volcanoes National Park (Fig. 1). In our model, the mosquito dynamics at a study site are a function of the site-specific daily temperature, daily rainfall, and the availability of larval mosquito habitat [7]. We fit thin plate smoothing splines to weekly temperature and rainfall data (1980 to 2004) for weather stations on Hawaii. From these spline surfaces, we predicted weekly temperatures and rainfall for our study sites. Average daily temperatures were estimated using interpolation. A random amount of the total weekly rainfall was assigned among days in a week to obtain daily rainfall. Climate methods and details for our study sites are described in Samuel et al. [7] and Hobbelen et al. [23].We used daily temperature and rainfall from 1980–2003 for each study site as the “baseline climate” with mean annual temperatures between 16.1–17.4°C (Table 1). Temperatures were highest in July or August and lowest in January and differed between 5.2–5.6°C. Rainfall was highest during the fall (Oct–Dec) and lowest in the spring (Apr–Jun). Dry days in a year (<5 mm rainfall) varied between 72–86%, while the number of heavy rainfall events (>255 mm during three days) ranged from 2.5–4 (Table 1).

**Table 1:** Characteristics of the study sites.

Site name	Altitude (m)	Mean annual temperature (°C)	Average yearly rainfall (mm)	% dry days in a year	Number of heavy rainfall events in a year	Cavity density (#/km^2^_)_	% cavities created by feral pigs	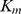 (♀ mosquito larvae/km^2^)
Waiakea	885	17.2 (0.9)	4081 (1017)	86 (5.3)	4.0 (4.1)	2751	100	162309
Cooper	1024	17.1 (0.3)	2816 (738)	81 (6.4)	2.5 (2.4)	3733	100	220247
Crater	1177	17.4 (0.3)	2095 (574)	72 (7.6)	1.5 (2.0)	432	0	25488
Pu'u	1247	16.1 (0.6)	3033 (791)	81 (6.4)	2.9 (2.7)	118	0	6962

For each site, the climate during the years 1980–2003 was estimated from field data using the program ANUSPLIN (see text). The climate data represent averages for the years 1980–2003. Values in brackets indicate standard deviations of the yearly averages. “Cavity” is used as a general term for the breeding habitat of mosquitoes and may consist of rock holes, man-made habitat, and cavities created by feral pigs. Cavity density and source (pig vs. other) were determined by field surveys. Method used to estimate carrying capacities of mosquito larvae based on the cavity density are described in Samuel et al. (2011).

We surveyed our 4 study sites for feral pig activity and larval mosquito habitat to determine the relationship between numbers of feral pigs and abundance of mosquito larvae. Most (82%) larval mosquito habitat and associated larval mosquitoes (Table 1) were in tree fern cavities created by pigs. Remaining habitats included rock holes, tree holes, ground pools, and artificial containers. The density of tree fern cavities is proportional to pig density (D. LaPointe, unpublished data) and we only found tree-fern cavities at the Cooper and Waiakea study sites. Cavities were absent at the Crater and Pu'u study sites where feral pigs had been eradicated (Table 1).

### Simulation model

**Figure 2:**
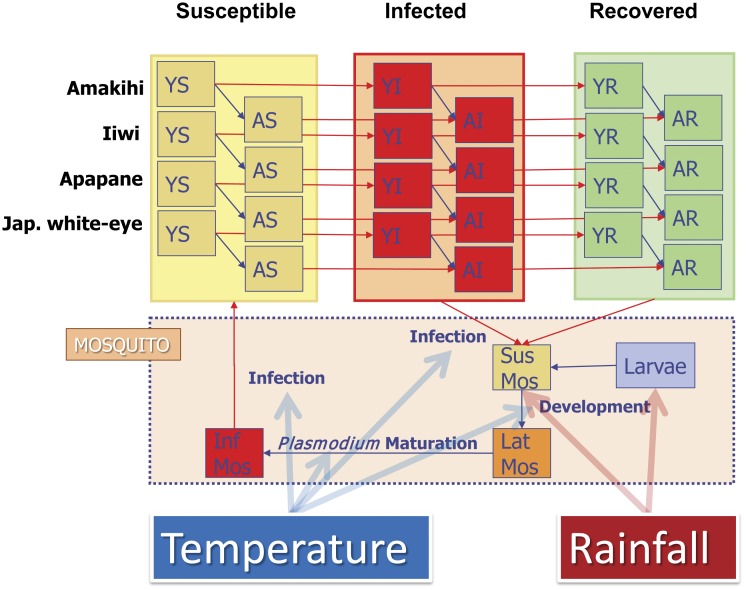
Malaria-forest bird epidemiological model. Mosquitoes develop from Larvae to susceptible adults (Suc Mos). They become infected, but not infectious (Lat Mos) after feeding on acutely infected (YI/AI) or chronically infected (YR/AR) birds. Once malaria parasites mature in a latent mosquito it becomes infectious (Inf Mos) to susceptible (YS/AS) birds. Temperature and rainfall affect the rates of mosquito development (Larvae→Sus Mos), malaria maturation (Lat Mos→Inf Mos), and infection.

Our simulation model [7] is based on the population dynamics of female Southern House mosquitoes (Culex quinquefasciatus), three endemic Hawaiian honeycreepers which represent the spectrum of malaria susceptibility/tolerance; the Hawaii Amakihi, Apapane (*Himatione sanguinea sanguinea*), and Iiwi (*Vestiaria coccinea*). The model also includes the Japanese White-eye (*Zosterops japonicus*) to represent introduced species, which typically have lower prevalence and intensity of infection with malaria [7]. Other native and introduced bird species were not considered in our model due either to low prevalence of infection or low abundance in our study sites. The general model features (Fig. 2) are described below; however, equations, parameter definitions and values, and other details are provided in Samuel et al [7].

Mosquitoes and all bird species are divided into immature and adult stages. Adult mosquitoes are subdivided into susceptible, latent, and infectious disease stages. Native juvenile (susceptible when hatched) and adult birds are subdivided into susceptible, acutely infected, and recovered stages. Acutely infected birds have a high parasitemia [24] and suffer from high disease mortality which varies by species [7]. Recovered - native birds that are able to survive infection are immune to subsequent infection; however, they remain chronically infected and able to transmit malaria parasites to mosquitoes [6] with only slightly lower probability than the acutely infected stage (C. Atkinson, unpublished data). Japanese White-eyes (and most introduced species) have a short time period when they are infectious to mosquitoes [7].

Mosquito dynamics are driven by rainfall and temperature [4], [5], [7]. Warmer temperatures shorten the development time for immature mosquitoes, reduce the length of the gonotrophic cycle in reproducing females, and increase the development rate of malaria parasites in mosquitoes [4], [7], [10]. Droughts (number of consecutive days with ≤5 mm rainfall; Table 1) and heavy rainfall events (>255 mm in three days; Table 1) decreased the survival of larval and adult mosquitoes, respectively [4]. We assumed female mosquitoes lay 100 female eggs during each gonotrophic cycle [4]. Larval mosquito carrying capacity was modeled as a single aggregate for all larval habitat within each study site (Table 1), and was not partitioned by individual cavities. We also assumed that reductions in larval habitat were long-term and therefore larval habitat was stable (equal additional and removal of larval habitat). Bird demographics are not directly affected by temperature and rainfall. However, demographics differ between elevations because biotic factors (which are influenced by climate) like food abundance and habitat differ by elevation and affect the seasonal breeding cycle. Bird density, which is limited by carrying capacity, was estimated from field studies [7]. We assume no interspecific competition among bird species in our model, which corresponds with previous findings for native species [25]. Although Japanese White-eyes may be an important competitor with native species [25]–[27]; the demographic impacts are limited and/or controversial [28]. Our model assumes malaria transmission between birds and mosquitoes is frequency dependent [29] and the probability of transmission from an infectious mosquito to a susceptible bird is 0.98 per successful mosquito bite [7].

### Model simulations

We modeled the potential impact of reducing larval habitat for two study sites where pig-created cavities provide an important habitat for mosquitoes (Cooper and Waiakea). We modeled the potential impact of Amakihi establishment at all four study sites. Initial conditions for our simulations used predicted bird densities in the absence of malaria [7], no infected birds, 100 infectious adult mosquitoes per km^2^, and no mosquito larvae. Simulations were then conducted for a 24 year period (1980–2003) using historical weather conditions (see above). Mosquito dynamics typically became stable within a few years regardless of the initial densities or elevation [7]. We evaluated model results based on annual mosquito densities for the last three (2001–2003) simulated years, because mosquitoes varied depending on seasonal and annual weather conditions. We used the last simulated year (2003) for bird densities which were generally stable to slowly declining near the end of the simulation. To determine the effect of reducing mosquito larval habitat, we simulated reductions in mosquito larvae habitat of 0 to 100% in steps of 5%. To model the effect of malaria-tolerant Amakihi, we assumed all Amakihi in the initial simulated population had the lower disease mortality rates found in low-elevation birds [7] (C. T. Atkinson, unpublished). Because these Amakihi are malaria-tolerant compared to conspecifics at other elevations, they are more likely to survive infection, creating a larger number of chronically infected birds which serve as a reservoir for malaria transmission to other susceptible honeycreepers.

To assess the potential effects of different weather patterns caused by variation in elevation and topography across the Hawaiian landscape, we modeled both conservation strategies under five climate scenarios derived from our baseline climate (1980–2003) data at study site Cooper (Table 1). Three different rainfall scenarios were simulated for the annual mean temperature of 17°C at Cooper (Table 1): 1) average site-specific annual rainfall of approximately 3000 mm, 2) lower annual rainfall of 2000 mm, and 3) higher annual rainfall of 4000 mm. Two different temperature scenarios were simulated for the average annual rainfall of 3000 mm: 1) a lower annual temperature of 15.5 and 2) a higher annual temperature of 18.5°C. All model simulations were performed in Matlab version 7.5.0 using solver ode45 with default settings [30].

## Results

### Mosquito density and prevalence

**Figure 3:**
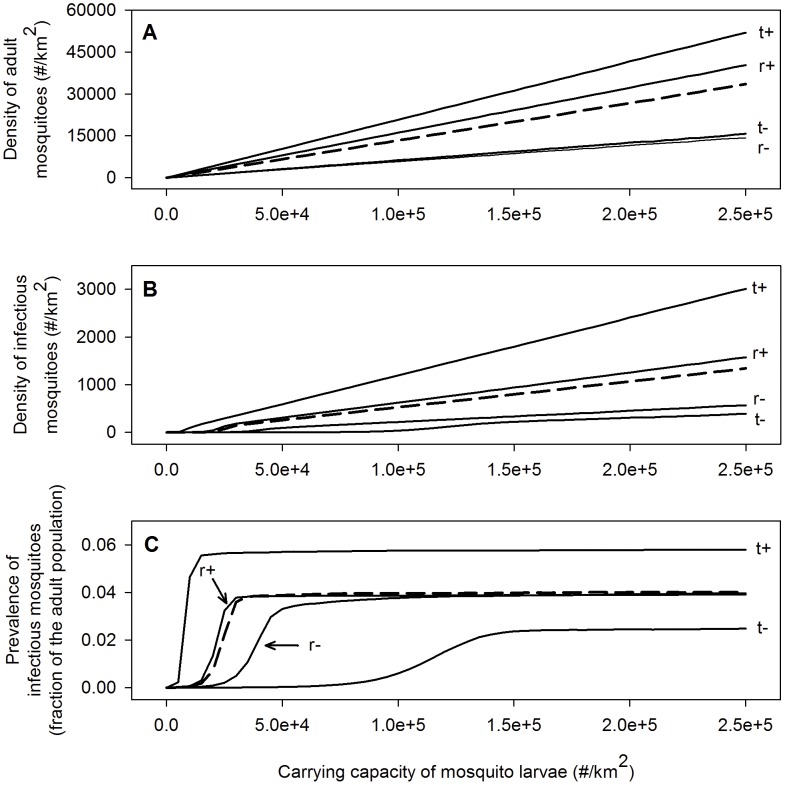
The mean annual densities of adult and infectious mosquitoes and the mean annual prevalence of infectious mosquitoes as a function of larval the carrying for different mid-elevation climate scenarios. The dashed line represents a climate with a mean annual temperature of 17°C and an average yearly rainfall of 3000 mm. The other climate scenarios are derived from this scenario by increasing or decreasing the daily temperatures and rainfall. The mean annual temperatures and average yearly amounts of rainfall for these scenarios are 15.5°C and 3000 mm (t−), 18.5°C and 3000 mm (t+), 17°C and 2000 mm (r−) and 17°C and 4000 mm (r+).

In mid-elevation forests with feral pigs, our model predicts that density of adult and infectious mosquitoes change in proportion to the carrying capacity for larval mosquitoes and in response to climate (Fig. 3). For baseline climate (average temperature and rainfall from 1980–2003), simulated prevalence of infectious mosquitoes stabilizes at a low rate despite increases in larval habitat and mosquito density, suggesting mosquito infection rates may reach an equilibrium that depends on the biting rate, probability of transmission from birds to susceptible mosquitoes, and parasite development rates. Climate scenarios with higher temperature or rainfall increase the density of adult and infectious mosquitoes (Fig. 3). Temperature has the largest effect on mosquito abundance and infectious mosquitoes. Higher (or lower) temperatures increase (decrease) the maximum prevalence of infectious mosquitoes and decrease (increase) the larval carrying capacity required to achieve this threshold (Fig. 3). In contrast, rainfall has limited effect on the prevalence of infectious mosquitoes.

### Mosquito control

**Figure 4:**
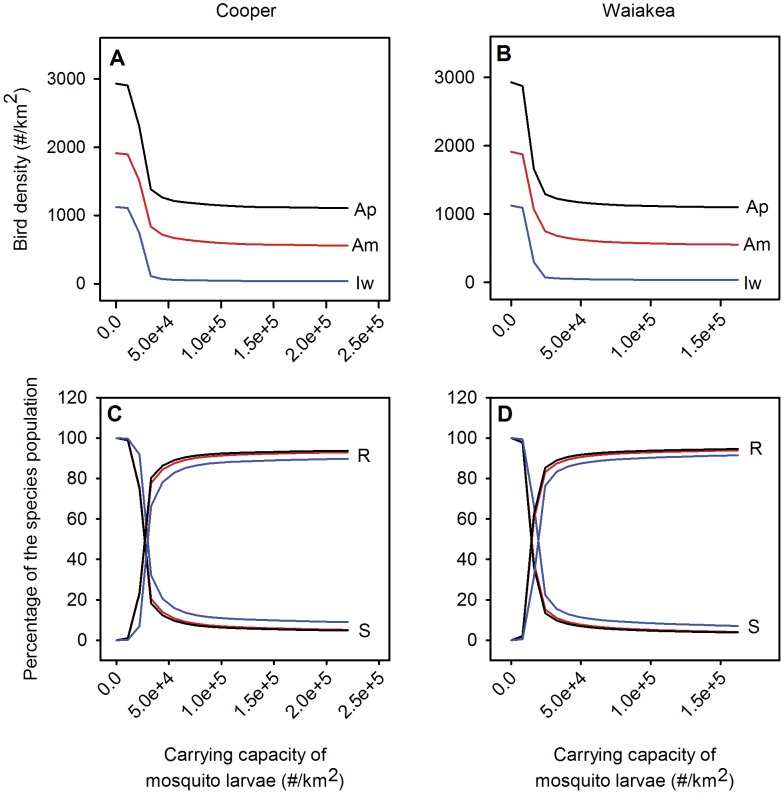
The mean annual densities of three Hawaiian honeycreepers and the mean annual percentages of their populations in the susceptible (S) and recovered (R or chronically-infected) stages as a function of larval carrying capacity for mid-elevation sites Cooper and Waiakea. Am = Amakihi (red line), Ap = Apapane (black line), and Iw = Iiwi (blue line).

**Figure 5:**
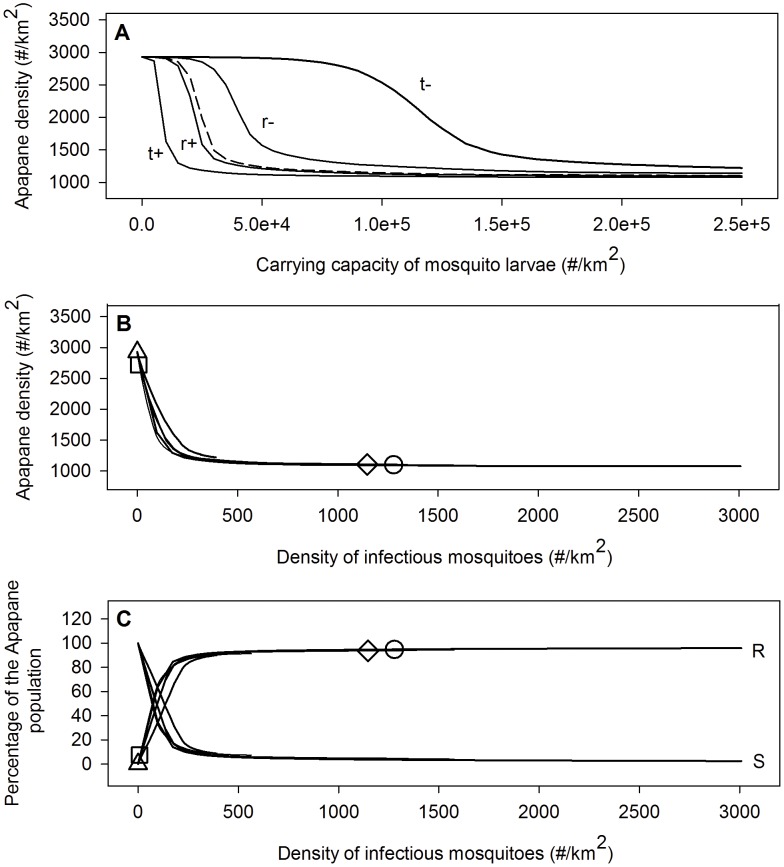
The mean annual densities of Apapane at mid-elevation as a function of larval carrying capacity and density of infectious mosquitoes. The bottom graph shows the percentage of Apapane in the susceptible (S, dotted line) and recovered stage (R, solid line) as a function of the density of infectious mosquitoes for different climate scenarios. The dashed line represents a climate with a mean annual temperature of 17°C and an average yearly rainfall of 3000 mm. The other climate scenarios are derived from this scenario by increasing or decreasing the daily temperatures and rainfall (see text). The mean annual temperatures and average yearly amounts of rainfall for these scenarios are 15.5°C and 3000 mm (t−), 18.5°C and 3000 mm (t+), 17°C and 2000 mm (r−) and 17°C and 4000 mm (r+). The markers in the two bottom figures indicate values for the biocomplexity field sites: circle = Waiakea, diamond = Cooper, square = Crater and triangle = Pu'u.

Our simulations indicate little improvement in honeycreeper abundance until a threshold was reached at low larval carrying capacity (Fig. 4). The threshold corresponds to the removal of ≥80% of the larval habitat for Cooper and Waiakea with predicted densities near 300 infectious mosquitoes/km^2^. Once this threshold was reached, further reductions produced dramatic increases in honeycreeper populations. These simulated population increases were accompanied by large increases in the percentage of susceptible and large decreases in the percentage of chronically-infected individuals (Fig. 5), indicating that reduction in disease transmission was the underlying driver. Changes in temperature had a stronger effect than changes in rainfall in determining the relationship between larval carrying capacity and changes in native bird populations (Fig. 5). Higher temperature, and to a smaller extent rainfall, meant that mosquito control by reducing larval carrying capacity was less effective (Fig. 5). These patterns were similar for all three honeycreeper species (data not shown) and increased abundance was accompanied by a corresponding increase in susceptible birds and a decline in chronically-infected birds (Fig. 4). Mosquito control efforts via reduction of larval habitat was more successful in areas where temperature and/or rainfall are lower or where available larval habitat was lower, conditions that common in higher elevation forests.

Weather patterns interact with the larval habitat reduction needed to enhance bird populations (Fig. 5). Effective mosquito control by larval habitat reduction corresponded to densities of <300 infectious adult mosquitoes/km^2^,, which was generally robust to differences in weather patterns, study sites, and species of honeycreeper. These results indicate that climate (especially temperature) is an important driver of mosquito dynamics, but density of infectious mosquitoes is the primary driver of malaria transmission to susceptible Hawaiian birds.

### Disease-tolerant Amakihi

Our model suggests that establishment of malaria-tolerant Amakihi would have little effect on total adult mosquito density (<1% change) in mid-elevation forests, but the density of infectious mosquitoes increased by 25% at Cooper, 26% at Waiakea, and 303% at Crater. At Pu'u, the density of infectious mosquitoes was <1/km^2^ after establishment of malaria-tolerant Amakihi. Presence of malaria-tolerant Amakihi in mid-elevation forests increased the predicted density of Amakihi by >900 birds/km^2^ at Cooper and Waiakea, while Apapane and Iiwi were not additionally affected (Table 2) because transmission rates at these sites were already high. At Crater, Amakihi density did not change, but reduced the predicted density of Apapane and Iiwi by 13% and 22%, respectively (Table 2). At Pu'u, where predicted mosquito abundance and density of infectious mosquitoes were low, malaria-tolerant Amakihi did not affect the density of other native birds (Table 2). The predicted prevalence of chronically-infected native birds, which provide a reservoir for malaria transmission, remained small at Pu'u (≤0.01%), stayed the same (>80%) at Cooper and Waiakea, but increased from 9 to 27% at Crater.

**Table 2:** The change in the mean annual model estimated densities of three Hawaiian honeycreeper species due to the translocation of disease-resistant Amakihi from low elevation to the mid-elevation sites Cooper, Crater, Waiakea and Pu'u.

	Density before translocation (#/km^2^)	Density after translocation (#/km^2^)	Change due to translocation (#/km^2^)
*Amakihi*			
Cooper	557	1522	965
Crater	1766	1792	26
Waiakea	552	1521	969
Pu'u	1911	1912	1
*Apapane*			
Cooper	1106	1104	−2
Crater	2710	2355	−355
Waiakea	1100	1100	0
Pu'u	2928	2928	0
*Iiwi*			
Cooper	37	37	0
Crater	996	772	−224
Waiakea	36	36	0
Pu'u	1122	1122	0

**Figure 6:**
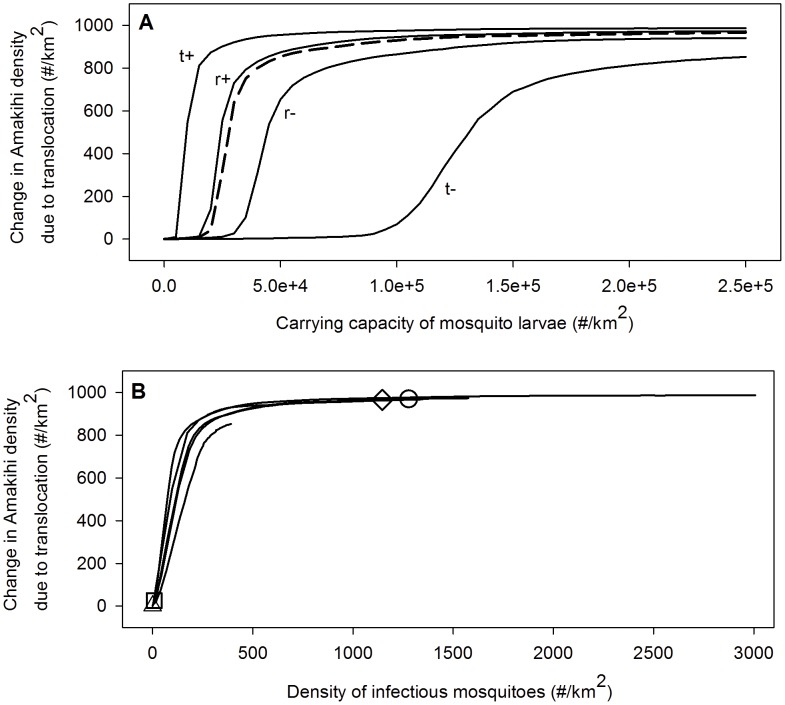
The change in mean annual densities of Amakihi at mid-elevation due to the translocation of disease-resistant Amakihi as a function of larval carrying capacity and density of infectious mosquitoes for different mid-elevation climate scenarios. The dotted line represents a climate with a mean annual temperature of 17°C and an average yearly rainfall of 3000 mm. The other climate scenarios are derived from this scenario by increasing or decreasing the daily temperatures and rainfall. The mean annual temperatures and average yearly amounts of rainfall for these scenarios are 15.5°C and 3000 mm (t−), 18.5°C and 3000 mm (t+), 17°C and 2000 mm (r−) and 17°C and 4000 mm (r+). The markers in the bottom figure indicate the mean annual bird densities at the biocomplexity field sites: circle = Waiakea, diamond = Cooper, square = Crater and triangle = Pu'u.

Predicted changes in malaria-tolerant Amakihi depended on the density of infectious mosquitoes (Fig. 6). Climate and larval carrying capacity, which influence the predicted density of infectious mosquitoes, have an important effect on Amakihi. Amakihi density increased most for climate scenarios with higher temperature (Fig. 6) and was greatest when infectious mosquitoes were above the 300/km^2^ threshold, indicating that less tolerant Amakihi populations at these sites were limited by malaria. In these simulations the proportion of chronically-infected Amakihi exceeded 85–89%, indicating high rates of disease transmission.

Predicted Apapane and Iiwi population responses to malaria-tolerant Amakihi depended on the density of infectious mosquitoes. Apapane and Iiwi densities were generally unaffected in forests where malaria transmission was either very low (Pu'u) or high (Waiakea and Cooper). At high transmission sites, malaria infection had already reached a high level so further increasing the reservoir of chronically-infected birds had little effect. At low transmission sites, predicted Amakihi density was not affected because there was little malaria impact and bird communities in these forests were unaffected. Apapane and Iiwi populations declined in forests where the density of infectious mosquitoes was below the threshold needed to enhance native bird populations (e.g., Crater). In these forests small increases in density of infectious mosquitoes increased rates of disease transmission which benefited malaria-tolerant Amakihi, but were detrimental to susceptible Apapane and Iiwi.

## Discussion

Avian malaria has a significant population impact on susceptible Hawaiian honeycreepers and disease transmission by mosquito vectors is likely a primary factor contributing to the decline and restricted distribution of native Hawaiian forest birds [2], [6], [7]. We used a computer simulation model of this complex disease system [7] to evaluate how two proposed conservation strategies might affect bird and mosquito populations. One of these management actions was designed to control mosquito populations by reducing the abundance of mosquito larval habitat. The second was to establish malaria-tolerant Amakihi populations in mid-elevation forests to increase abundance of this native species. In the future it seems likely that these malaria-tolerant Amakihi will naturally disperse to mid-elevation forests; therefore, assessment of the potential long-term effects of this migration is also relevant to future avian conservation.

### Mosquito larval control

Our modeling indicates that infectious mosquitoes need to be reduced below a threshold (predicted <300 individuals/km^2^) before mosquito control benefits mid-elevation honeycreepers. For infectious mosquito densities above this threshold, most surviving birds are predicted to be chronically-infected with malaria, and therefore a disease reservoir for susceptible birds. Although both rainfall and temperature affect the relationship between larval habitat and malaria transmission, model results indicate that the density of infectious mosquitoes was the key factor influencing transmission and bird abundance (Fig. 5). In forests with limited larval habitat (e.g., Crater and Pu'u), mosquito control is unlikely to be effective because mosquito density and malaria transmission is already limited. In other forests, reduction in larval habitat can substantially reduce mosquito density. In many mid-elevation forests (e.g., Cooper and Waiakea) with high, infectious mosquito densities, nearly complete larval control will be needed before infectious mosquitoes decline sufficiently to reduce malaria transmission and benefit bird populations. Our results indicate that near eradication or continual control of mosquito larval habitat may be a potential, although challenging, long-term solution to recovery of Hawaiian birds in mid-elevation forests.

On the Island of Hawaii, cavities created by feral pigs in native tree ferns are an important larval habitat for mosquitoes, especially in mid-elevation forests (e.g. Cooper and Waiakea; Table 1) with abundant native tree ferns [16]. At these sites, removal of feral pigs could reduce the amount of larval habitat for mosquitoes and decrease the impact of avian malaria on endemic Hawaiian honeycreepers. However, because the density of tree fern cavities is approximately proportional to the pig density (D. LaPointe, unpublished data), almost complete pig removal is needed to achieve larval habitat reductions that benefit native birds. Iiwi populations have been observed in some mid-elevation forest sites where pigs have been successfully removed and larval cavities have been substantially reduced, providing support for the benefit of pig management (Pu'u, Table 1).

Several limitations in our analysis should be considered in evaluating mosquito control strategies. Our simulations were based on reductions in total larval carrying capacity and not reductions based on the number of cavities or cavity-specific larval productivity. If the actual distribution of larval capacity is heterogeneous, then specific strategies that depend on the size distribution of larval cavities or on larval productivity may be required to achieve >80% reductions. Model sensitivity analyses [7] showed that mosquito demographics (adult mortality, gonotrophic cycle length, and larval mortality rate) were important predictors of bird population responses, suggesting integrated mosquito control strategies which simultaneously reduce larval habitat, increase mortality on adult or larval mosquitoes, and reduce the feeding rate of mosquitoes on birds might provide effective strategies which merit further evaluation for many mid-elevation forests.

Migration of infected mosquitoes up to 3 km from adjacent unmanaged areas [31] may reduce the efficiency of mosquito control strategies on smaller isolated areas. Bird movement out of mosquito control areas may also produce additional malaria infection and reduce effectiveness. Better information on mosquito and bird movements would help determine the appropriate size for mosquito control zones. Transmission rates may result in differential selection for virulent strains of avian malaria [32], [33], but we did not consider differential virulence in response to transmission rates. In some of the mid-elevation forests, mosquito larval habitat is closely tied to riparian systems, but current understanding of mosquito dynamics and therefore control strategies in these systems is limited. Finally, similar responses among honeycreeper species (Fig. 5) to removal of larval habitat and therefore density of infectious mosquitoes (Fig. 3), likely occurs because our model assumes that mosquitoes have equal preference for feeding on all honeycreeper species. Further research to determine how mosquito feeding preferences or bird defensive behavior influences malaria transmission is needed.

### Malaria-tolerant Amakihi

Our model indicates malaria-tolerant Amakihi could increase the abundance of infectious mosquitoes in some mid-elevation forests and therefore decrease densities of more susceptible honeycreepers. The negative impact of malaria-tolerant Amakihi on other honeycreepers should be highest at sites where density of infectious mosquitoes is below a predicted threshold of 300/km^2^ (e.g., Crater). Above this threshold, an increase in the density of infectious mosquitoes from malaria-tolerant Amakihi does not significantly increase the impact of avian malaria on bird populations (Fig. 5). Overall, it appears that movement of malaria-tolerant Amakihi into mid-elevation forests will have positive or neutral benefits to Amakihi populations and either neutral or negative impacts on other native honeycreeper species. However, mosquito population controls designed to benefit native birds could be adversely affected by malaria-tolerant Amakihi which provide a larger reservoir of chronically-infected birds capable of transmitting malaria to mosquitoes and then to susceptible native species. In some forests, malaria-tolerant Amakihi could require an increase in mosquito control efficacy.

Whether translocation of Amakihi would succeed depends on many factors [34], [35] including habitat quality, competitors, reproductive traits, location of release within the species range, number of animals released, and program length. To maximize the likelihood of success, the heterozygous offspring from disease-tolerant and non-tolerant Amakihi should also be tolerant, tolerance should have minimal cost to fitness, and gene loss from drift should be low. We did not evaluate the probability for a successful translocation program, the length of time and efforts required, or the potential for low-elevation Amakihi to naturally move to mid-elevation forests. Nor did we consider whether malaria-tolerant Amakihi in mid-elevation forests would result in increased pathogen virulence; although there is no current evidence of higher pathogen virulence in low-elevation forests dominated by these birds.

### Climate implications

In Hawaii, malaria infection patterns are driven by the effects of temperature and rainfall on mosquito dynamics across an elevational gradient, seasonal weather patterns, and availability of larval habitat [7]. Projected climate warming of 2–3°C by 2100 [36], [37] will certainly increase the need for conservation measures to reduce malaria transmission, but will also increase the challenge in developing successful strategies. Under current climate patterns, high-elevation forests serve as a disease-free refuge for susceptible birds. However, future climate warming will likely reduce these high-elevation refuges for Hawaiian forest birds [8], [9]. Our modeling results suggest that climate warming will make mosquito control in mid-elevation forests more difficult by requiring nearly complete elimination of larval habitat to benefit native bird populations (Fig. 5). In general, our results indicate that further evaluation is needed to understand the specific long-term impacts of climate warming on malaria transmission in Hawaii and to develop effective management strategies to mitigate climate change.

## Conclusions

Even low densities of infectious mosquitoes appear sufficient to produce high rates of malaria infection in native Hawaiian birds. In part, these high rates of malaria transmission likely result from favorable climatic conditions, abundant larval mosquito habitat, and efficient transmission of the malaria parasite between mosquito vectors and avian hosts. Native birds that survive malaria infection acquire immunity, but also become an effective reservoir for malaria transmission to mosquitoes and then to susceptible Hawaiian birds. Reducing the amount of larval habitat for mosquitoes by feral pig management or other alternatives provides one potential strategy to help control malaria impacts and would likely be most beneficial in mid-elevation forests. Establishment of malaria-tolerant Amakihi to mid-elevation forests benefits Amakihi populations, but could negatively impact other honeycreepers depending on the larval habitat and malaria transmission. Our evaluation uses an ecological model that provides a simplified representation of the Hawaiian forest bird malaria system which appears to provide realistic, but somewhat uncertain predictions of mosquito, bird, and malaria patterns in Hawaii [7]. Despite these limitations, our model provides a valuable decision support tool for evaluating alternative conservation actions which would be difficult to implement and/or extremely costly for such a complex ecological system [22]. Because of these limitations we recommend that modeling be used to generate hypothesized responses to management actions. This provides the framework for an adaptive management process [38], [39] that can be used in conjunction with experimental studies and management actions that can verify model predictions and improve model performance prior to implementation of costly large-scale conservations actions [1].
