# Correction: Modeling Future Conservation of Hawaiian Honeycreepers by Mosquito Management and Translocation of Disease-Tolerant Amakihi

**DOI:** 10.1371/annotation/d626ea7a-acd3-41a4-b6c5-83e79ead02d7

**Published:** 2013-06-04

**Authors:** Peter H. F. Hobbelen, Michael D. Samuel, Dennis A. LaPointe, Carter T. Atkinson

There is a sentence missing from the Funding Statement. The following is the correct Funding Statement:

This work was supported with funding from the National Science Foundation (DEB 0083944) and the U.S. Geological Survey Wildlife and Invasive Species Programs. The Department of Forest and Wildlife Ecology at the University of Wisconsin provided funds for publication. The funders had no role in study design, data collection and analysis, decision to publish, or preparation of the manuscript. 

